# Personalized recurrence risk prediction in early-stage breast cancer through an integrative mathematical model based on MammaPrint®, radiotherapy, phenotype, and clinicopathological factors

**DOI:** 10.1007/s12094-025-04065-y

**Published:** 2025-10-03

**Authors:** J. Sánchez Mazón, A. de Juan Ferré, J. M. López Vega, C. López López

**Affiliations:** 1 Department of Medicine and Psychiatry, University of Cantabria, Santander, 39008 Spain; 2 Oncology Department, Marqués de Valdecilla University Hospital, Santander, 39008 Spain

**Keywords:** Recurrence, Breast cancer, MammaPrint, Radiotherapy

## Abstract

****Purpose**:**

To build a mathematical model with predictive capacity for the risk of recurrence in patients with early-stage breast cancer, based on four variables: the result of the MammaPrint®(MMP) test, postoperative radiotherapy (RT), tumor phenotype, and clinicopathological criteria. To estimate overall survival functions stratified by the dose of radiotherapy received.

****Methods**:**

A retrospective cohort of 156 patients with early-stage breast cancer was analyzed. Patients were classified according to their MammaPrint®genomic risk (ultralow, low, or high). Multivariate logistic regression using Firth’s method was employed to evaluate the risk of recurrence, adjusting for biologically effective dose (BED), molecular subtype, their MammaPrint®classification and clinico-pathologic features. Receiver operating characteristic (ROC) analysis was used to assess model discrimination. The Kaplan–Meier method was used to estimate overall survival (OS) functions. To assess statistically significant differences in survival between patient groups, the log-rank test was applied.

****Results**:**

The predictive model, incorporating BED, genomic risk, molecular phenotype, and clinico-pathological classification, showed good calibration and discrimination (AUC: 0.755). The evaluation of OS according to the different BED levels provides clearer results regarding the clinical benefit of radiotherapy. This study reports statistically significant differences when comparing the group without radiotherapy (BED = 0 Gy) to the low-dose group (BED < 60 Gy), with a *p*-value of 0.0475.

****Conclusion**:**

The predictive model fitted using Firth’s penalized logistic regression demonstrated an adequate discriminative ability (AUC = 0.755). MMP was the variable with the greatest weight, followed by RT. These variables allow for a more accurate prediction of recurrence risk than traditional clinicopathological factors, supporting their value in the personalization of treatment. This study reports statistically significant differences when comparing the group without radiotherapy (BED = 0 Gy) to the low-dose group (BED < 60 Gy), with a *p*-value of 0.0475.

## Introduction

Breast-conserving surgery followed by whole-breast irradiation remains the standard of care for most patients with early-stage breast cancer [1, 2]. Radiotherapy has been shown to significantly reduce the risk of local recurrence and contribute modestly to improvements in overall survival [3]. However, concerns about long-term toxicity, quality of life, and overtreatment have fueled interest in personalized treatment approaches that safely reduce therapy intensity without compromising efficacy [2]. Gene expression assays have emerged as valuable tools for stratifying breast cancer patients beyond traditional clinico-pathologic parameters. Among these, the MammaPrint®70-gene signature has been validated in several large-scale trials, including the MINDACT study, to guide decisions regarding adjuvant systemic therapy [4, 5]. More recently, attention has turned to the potential utility of these genomic tools in informing radiotherapy decisions [6]. In particular, the ultralow-risk category within the MammaPrint®classification identifies tumors with extremely favorable biological behavior. Esserman et al. reported excellent outcomes in this group even in the absence of systemic therapy, suggesting that such patients may also derive minimal benefit from adjuvant radiotherapy [7]. Subsequent analyses have supported the prognostic value of this category in both node-negative and hormone receptor-positive disease [8]. Despite this promising evidence, radiotherapy indications still rely predominantly on age, tumor size, margin status, and histologic grade, without formally incorporating genomic risk profiles [9]. Furthermore, few studies have integrated dosimetric parameters, such as the biologically effective dose (BED), with genomic and phenotypic data to develop individualized models for predicting locoregional recurrence risk [10]. The aim of this study was to investigate whether the MammaPrint®risk classification, combined with molecular phenotype, standard clinico-pathological features, and radiation dose characteristics, could serve as a predictive model for locoregional recurrence in a cohort of early-stage breast cancer patients treated with breast-conserving surgery. We hypothesized that patients with an ultralow-risk genomic profile receiving adequate BED would demonstrate extremely low recurrence risk, supporting safe omission of radiotherapy in selected cases.

## Methods

### Study design and population

A retrospective observational study was conducted involving 156 patients diagnosed with early-stage invasive breast cancer between 2013 and 2018. Inclusion criteria addressed the following variables: patients with a histopathological diagnosis of breast cancer, treated with either conservative or radical surgery, with or without radiotherapy, and a minimum follow-up period of 5 years after diagnosis. As exclusion criteria, only patients with triple-negative hormone receptor status (negative ER, negative PR, and negative HER2) or missing follow-up information were excluded.

### Analyzed variables

A summary of the study variables and their corresponding values is presented in Table 1.

**Table 1:** Study variables and corresponding values

Variable	Value
MMP	ULow
	Low
	High
CLINPAT	Low
	High
PHENOTYPE	Luminal A
	Luminal B
RT	$\textrm{BED} = {0\,\textrm{Gy}}$
	$$\textrm{BED} \le {60\,\textrm{Gy}}$$
	$$\textrm{BED} > {60\,\textrm{Gy}}$$

1. **MMP:** MammaPrint®test result. This variable is based on the gene expression profiling of 70 genes associated with recurrence risk. According to the expression patterns, tumors are categorized into three types: low risk when the estimated probability of recurrence is approximately 10 %–15 % within 10 years after diagnosis; ultra-low risk when the probability is below that threshold; and high risk when the recurrence probability is around 30 %–35 % at 10 years. This is therefore a trichotomous variable.
2. **CLINPAT:** Clinicopathological criteria. This variable was constructed from surveys completed by medical oncologists from several hospitals. Each oncologist was presented with 20 randomly selected cases from the database, providing only clinicopathological information. They were asked to assign a score of either *high risk* (requiring chemotherapy) or *low risk* (not requiring chemotherapy). As such, this is a dichotomous variable.
3. **PHENOTYPE:** Histological subtype. Defined as a dichotomous variable that can take the values *luminal A* or *luminal B*. The former typically has a better prognosis and is more responsive to hormone therapy, while the latter is considered more aggressive and associated with a poorer prognosis. Classification was based on the latest St. Gallen consensus criteria [11].
4. **RT:** Radiotherapy treatment. All patients were evaluated for adjuvant radiotherapy following institutional protocols. Whole-breast irradiation was delivered using 3D-conformal or IMRT techniques. Total dose and fractionation schemes varied according to clinical criteria. The biologically effective dose (BED) was calculated for each patient using the linear-quadratic model ($\alpha /\beta = 4$ for breast tissue), and categorized into three groups: 0 Gy (No RT), $\le {60\,\textrm{Gy}}$, and $>{60\,\textrm{Gy}}$ (boost in the tumor bed).

### Statistical analysis

A binary logistic regression analysis was performed to estimate the mathematical function relating the probability of recurrence to the four variables of interest, with the aim of identifying which of them are most strongly associated with the risk of recurrence. Given that there are four covariates under study, at least 40 recurrence events would be required (10 per variable). Consequently, the application of standard logistic regression would lead to unstable estimates with excessively large standard errors, a situation commonly associated with the phenomenon of complete or quasi-complete separation. To mitigate these issues and obtain less biased estimates, Firth’s penalized logistic regression was employed. This method incorporates a penalty term based on Jeffrey’s prior information and has proven especially useful in low-event scenarios, as it reduces maximum likelihood bias and yields more realistic confidence intervals.

Furthermore, since all covariates are categorical, dummy coding was applied, selecting a reference or baseline category for each variable. In each case, the category representing the lowest clinical risk (or considered the baseline condition) was chosen as the reference. As a result, the reported coefficients represent the difference in the log odds of recurrence (and, when exponentiated, the odds ratios) for each category relative to the reference category, which is assumed to have no effect (OR = 1). This approach emphasizes the relative effects of each predictor and facilitates the interpretation of how recurrence odds change when shifting from the reference to alternative categories.

Additionally, a diagram was constructed to illustrate the relative contribution of each variable to the risk of recurrence. This visualization was based on the coefficients obtained from the fitted model. Each coefficient represents the effect of a specific category on the risk of recurrence, expressed as a change in the log-odds compared to a reference category. To assess the relative importance of the different factors, the absolute value of each coefficient was calculated, thereby capturing the magnitude of the effect regardless of whether it increased or decreased the risk. These values were then normalized by dividing each one by the largest absolute coefficient, allowing for a proportional comparison.

Finally, the model’s performance was evaluated using the Receiver Operating Characteristic (ROC) curve and the Area Under the Curve (AUC), applying bootstrapping techniques. This involved repeatedly resampling the dataset and computing the AUC in each iteration, in order to estimate the desired percentiles (2.5 % and 97.5 % ) and construct a 95 % confidence interval.

### Overall survival analysis

The Kaplan–Meier methodology was used to estimate survival functions, providing a clear visualization of the probability of survival over time for the entire patient cohort. Overall survival curves stratified by BED were constructed. Additionally, to assess statistically significant differences in survival between different patient groups, the log-rank test was applied.

## Results

A total of 156 patients were included in the study. Data were collected on local recurrence and disease-free survival according to genomic risk profiles (high and low). Radiotherapy treatment characteristics were also analyzed. The median follow-up was 6 years.

Table 2 shows the demographic characteristics of the patients. All were diagnosed with breast cancer between 2013 and 2018. The mean age at diagnosis was ($55.3 \pm 10.8$) years. Ages ranged from 28 to 81 years. A total of 46.8 % were younger than 55 years at diagnosis.

Half of the patients (50.6 %) were diagnosed through a breast cancer screening program. The remaining 49.4 % presented with symptoms. Regarding menopausal status, 34.6 % were premenopausal at diagnosis.

**Table 2:** Demographic data of the patients included in the study (n = 156)

Variable	Value
Mean age at diagnosis	$(55.3 \pm 10.8)$ years
Age range	28 to 81 years
Under 55 years	46.8 %
Diagnosed through a screening program	50.6 %
Symptomatic patients	49.4 %
Premenopausal	34.6 %

### Baseline characteristics of the patients

Table 3 presents the clinicopathological data of the tumors. Most tumors were classified as T1 or T2. Histological grading showed that 22.6 % of tumors were grade 1, 58.7 % grade 2, 15.5 % grade 3, and in 3.2 % the grade was unknown. Sentinel lymph node biopsy using the OSNA method (One Step Nucleic Acid Amplification molecular technique) identified the presence of lymph node micrometastases in 49 % of patients, mostly involving 1 to 3 positive nodes. Signs of lymphovascular or perineural invasion were observed in 55.4 % of cases.

Regarding the Ki67 proliferation index, 28.8 % of tumors had values below 20 %, while 60.3 % had values above this threshold. As for the histopathological diagnosis, 75.5 % were classified as invasive ductal carcinoma, 15.2 % as lobular carcinoma, and the remaining 3.3 % as mixed. Of all tumors, 26.5 % exhibited multifocality.

Concerning the molecular subtype (phenotype), all tumors analyzed were luminal. A similar distribution was found between Luminal A and Luminal B subtypes, accounting for 57 % and 42 %, respectively.

In terms of TNM staging, all patients had localized disease without distant metastases. Among those with lymph node involvement, none had more than three positive nodes. A total of 61.4 % of tumors were classified as T1.

**Table 3:** Clinicopathological characteristics of the tumors (n = 156)

Characteristic	Value	Percentage
Mean tumor size	$(21 \pm 12)\,{mm}$	
Less than 20 mm		54.6 %
Between 21 mm and 50 mm		43.2 %
Greater than 50 mm		2.2 %
Histological grade		
Grade 1		22.6 %
Grade 2		58.7 %
Grade 3		15.5 %
Unknown grade		3.2 %
Lymph node micrometastases		49 %
Lymphovascular or perineural invasion		55.4 %
Ki67 proliferation index		
Less than 20%		28.8 %
Greater than 20%		60.3 %
Histopathological diagnosis		
Invasive ductal carcinoma		75.5 %
Lobular carcinoma		15.2 %
Mixed		3.3 %
Multifocal nature		26.5 %
Fenotype		
Luminal A		57 %
Luminal B		42 %
Stage		
IA		36.2 %
IB		25.2 %
IIA		20.6 %
IIB		17.6 %

### Adjusted multivariable model

Table 4 provides a concise summary of the main results obtained from the penalized logistic regression model (Firth) and the evaluation of its discriminatory ability. It presents, on the one hand, the model coefficients for each predictor (including the intercept), expressed as odds ratios along with their 95 % confidence intervals and associated *p*-values; these data allow visualization of the direction and magnitude of the effect of each variable on the probability of recurrence. On the other hand, the table includes a section showing the overall performance of the model through the AUC obtained from the ROC curve, along with its confidence interval calculated using bootstrapping techniques, providing robust information essential for understanding the model’s estimation and stability. Table 4displays only the coefficients corresponding to the intercept and to the comparative categories of each variable.

**Table 4:** Results of the Firth logistic regression model and discriminative performance evaluation

Variable	Odds ratio (95% CI)	*p*-value
(Intercept)	0.021 (0.00014, 0.263)	0.0011
MMP Low	7.30 (0.79, 975.8)	0.0870
MMP High	8.11 (0.77, 1111.9)	0.0879
High CLINPAT risk	2.17 (0.51, 8.90)	0.3361
$\textrm{BED}<{60\,\textrm{Gy}}$	0.18 (0.022, 1.58)	0.1437
$\textrm{BED}>{60\,\textrm{Gy}}$	0.47 (0.13, 1.85)	0.2703
Luminal B	0.94 (0.25, 3.51)	0.9480
Discriminative performance:		
AUC	0.7554 (95% CI: 0.6498–0.8614)	

**Fig. 1:**
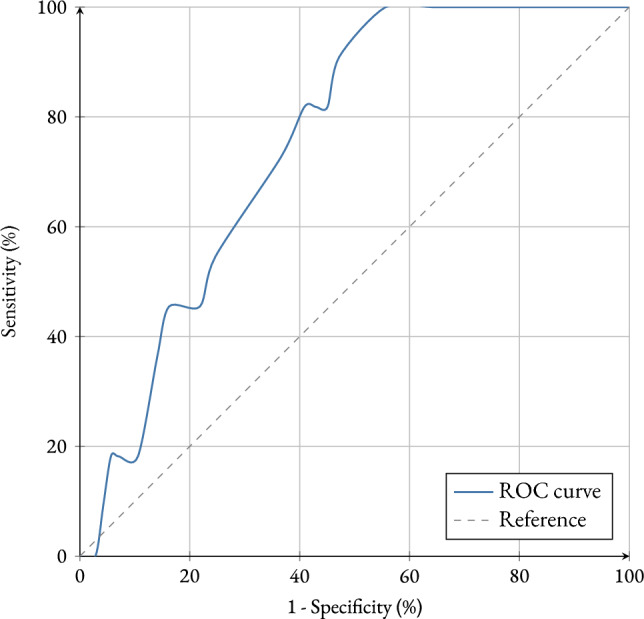
ROC curve showing the relationship between the false positive rate and the true positive rate at different classification thresholds. AUC = 0.7554

To assess the model’s ability to discriminate between patients who experienced recurrence and those who did not, the ROC curve was calculated based on the fitted model. Figure 1 provides a graphical representation illustrating the relationship between the false positive rate and the true positive rate as the classification threshold varies. In this study, the ROC curve coordinates were obtained and expressed as percentages, allowing a clear visualization of the model’s discriminative capacity.

The area under the curve (AUC) was 0.7554, with a 95 % confidence interval (0.6498–-0.8614) obtained through 1,000 stratified bootstrap replications. This AUC value indicates that the model has a good ability to distinguish between patients with and without recurrence, performing significantly better than a random classifier (AUC = 0.5). It is worth noting that a detailed analysis of the ROC curve, including the comparison of different thresholds and a more thorough interpretation of the AUC, will be presented in the following section. At this point, the aim is simply to present the graphical tool and the overall parameters that will serve as a reference for evaluating the predictive value of the model.

### Overall survival analysis

**Fig. 2:**
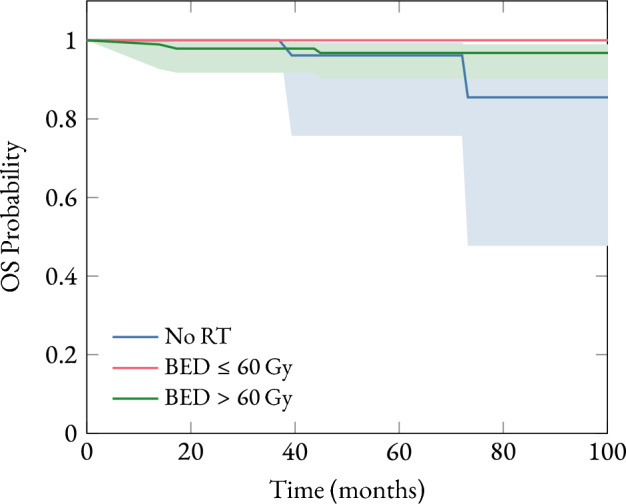
Kaplan–Meier survival function showing the estimated probability of overall survival (OS) stratified according to the levels of postoperative radiotherapy (RT) received. Shaded areas represent the ${95\,\mathrm{\%}}$ confidence intervals for the survival estimates. *p*-values: p($\textrm{BED}= {0\,\textrm{Gy}}$-$\textrm{BED}<{60\,\textrm{Gy}}$)=0.0475, p($\textrm{BED}={0\,\textrm{Gy}}$-$\textrm{BED}>{60\,\textrm{Gy}}$)=0.3016 y p( $\textrm{BED}>{60\,\textrm{Gy}}$-$\textrm{BED}<{60\,\textrm{Gy}}$)=0.3136

The overall survival (OS) analysis was performed using the Kaplan-Meier method, stratifying patients according to the biologically effective dose received. The results are presented in Fig. 2.

## Discussion

To perform the multivariate analysis, a penalized logistic regression model (Firth method) was used to identify factors independently associated with the risk of recurrence in the patient cohort. This model is particularly appropriate given the study characteristics, as the number of events was limited due to the good prognosis of the patients, making the use of conventional logistic regression unsuitable due to the phenomenon of complete or quasi-complete separation. The results obtained with this method are presented in Table 4. Based on the coefficients from the model, the multivariate logistic regression equation on the log-odds scale is expressed as:

1 $$\begin{aligned} \begin{aligned}&\log \left( \frac{p}{1-p}\right) =\; -3.8855 \\&\quad + 1.9888\,\mathrm {MMP(Low)} + 2.0928\,\mathrm {MMP(High)} \\&\quad + 0.6663\,\mathrm {CLINPAT(High)} \\&\quad - 1.4055\,(\textrm{BED}\le {60\,\textrm{Gy}}) - 0.7439\,(\textrm{BED}>{60\,\textrm{Gy}}) \\&\quad - 0.0432\,\textrm{Luminal B}\,. \end{aligned} \end{aligned}$$

Here, $-$3.8855 is the intercept, representing the log-odds of recurrence for the reference category (MMP ULow, CLINPAT Low risk, $\textrm{BED}={0\,\textrm{Gy}}$, and Luminal A PHENOTYPE). The term (1.9888.MMP Low) indicates that moving from the reference category (MMP ULow) to MMP Low increases the log-odds of recurrence by 1.9888. Similarly, (2.0928.MMP High) represents an increase of 2.0928 in the log-odds of recurrence when moving to the MMP High category. The remaining coefficients are interpreted analogously.

These results suggest relevant trends, although statistical significance was not reached due to wide confidence intervals, likely related to the limited number of recurrence events. Nevertheless, the findings provide preliminary evidence of the importance of the MMP result as a predictor of recurrence, showing notably high odds ratios for the MMP Low and High categories. This suggests that patients with higher-risk genomic profiles may have substantially increased odds of recurrence compared to those classified as MMP ULow.

The limited influence of variables such as CLINPAT, BED, and FENOTYPE in this multivariate model may be due to the insufficient sample size or the possibility that their effects are not independent once adjusted for the genomic information provided by the MMP test.

**Fig. 3:**
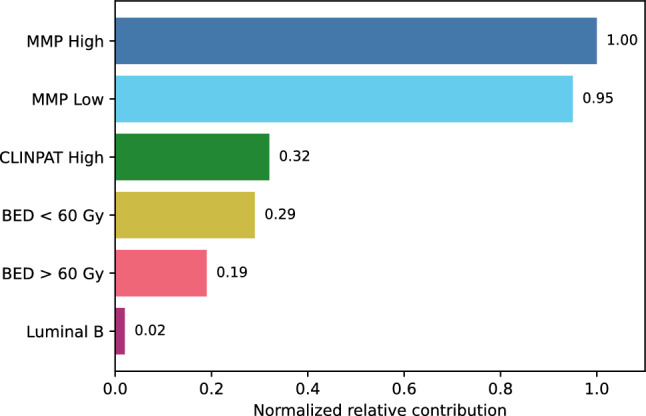
Relative contribution of each variable to the risk of recurrence, according to the adjusted multivariable logistic regression model. The magnitude of each effect is expressed in normalized values relative to the MMP High coefficient

Figure 3 shows the relative contribution of the variables included in the multivariable logistic regression model to the estimated risk of recurrence. The magnitude of each effect is expressed as normalized values with respect to the category with the greatest impact (MMP High) (see Table 4), enabling a comparative visualization of the influence of each factor on the clinical outcome.

As previously mentioned, this analysis uses as a reference those categories that are not represented in the graph, as they correspond to the model’s baseline categories. Specifically, the values are compared against: MMP ULow, $\textrm{BED} = {0\,\textrm{Gy}}$, CLINPAT low risk, and Luminal A phenotype.

The results show that the variables with the greatest impact on the increased risk of recurrence are those associated with the MMP classification, particularly in the Low and High groups, with a relative contribution of 95 % and 100 %, respectively, compared to MMP ULow. This reinforces the prognostic value of biological profiles, in line with recent evidence highlighting the importance of molecular characterization as a key risk factor [12].

Secondly, negative coefficients are observed for the levels $\textrm{BED} < {60\,\textrm{Gy}}$ and $\textrm{BED} > {60\,\textrm{Gy}}$, in comparison to the no-radiotherapy group ($\textrm{BED} = {0\,\textrm{Gy}}$). This suggests a beneficial effect of adjuvant radiotherapy, with a greater impact when the dose exceeds certain biologically effective thresholds. Although smaller than the effect of molecular profiling, this benefit suggests the therapeutic value of radiotherapy in reducing recurrence risk.

The CLINPAT High category contributes moderately (32 %), indicating that although clinicopathological variables remain relevant, their weight is lower than that of molecular and therapeutic factors. Lastly, the Luminal B category, compared to Luminal A, shows a marginal impact (2 %), suggesting that within the context of the adjusted model, its additional discriminatory power is limited.

These results support the usefulness of an integrated approach combining clinical, biological, and therapeutic information for individualized recurrence risk estimation.

The model’s discriminatory ability was further assessed using the ROC curve (see Fig. 1), obtaining an area under the curve (AUC) of 0.7554 (95 % CI: 0.6498–0.8614). This indicates that the model has a reasonably good ability to distinguish between patients who will experience recurrence and those who will not. Although not exceptionally high, this AUC represents a significant improvement over a random classifier (AUC = 0.5), highlighting the meaningful contribution of the included variables to recurrence prediction.

Therefore, the use of Firth’s penalized logistic regression appears methodologically appropriate in this context given the study’s limitations. This method corrects the bias of maximum likelihood estimates in settings with rare or sparse events and provides less biased estimates in cases of near-perfect separation.

Finally, the evaluation of OS according to different BED levels provides clearer insights into the clinical benefit of radiotherapy. This study reports statistically significant differences when comparing the group without radiotherapy (BED = 0 Gy) to those receiving low doses (BED < 60 Gy), with a p-value of 0.0475. This indicates a statistically significant improvement in overall survival when administering RT at doses below 60 Gy, compared to no RT. This finding is clinically relevant, as it suggests a clear survival benefit from radiotherapy even at moderate doses. In contrast, no statistically significant differences were observed when comparing patients who did not receive RT with those who received doses above 60 Gy (p = 0.3016). Similarly, no significant differences were found between high and low dose groups (p = 0.3136), suggesting that increasing the dose beyond a certain threshold (60 Gy) may not provide additional benefits in overall survival and may instead increase toxicity.

## Conclusion

This study aimed to build a mathematical model with predictive capacity for the risk of recurrence in patients with early-stage breast cancer, based on four variables: the result of the MammaPrint®test, postoperative radiotherapy, tumor phenotype, and clinicopathological criteria. The results suggest that the predictive model fitted using Firth’s penalized logistic regression demonstrated an adequate discriminative ability (AUC = 0.755). MMP was the variable with the greatest weight, followed by RT. These variables enable a more accurate prediction of recurrence risk compared to traditional clinicopathological factors, supporting their value in treatment personalization. This study reports statistically significant differences when comparing the group without radiotherapy (BED = 0 Gy) to the low-dose group ($\textrm{BED} \le {60\,\textrm{Gy}}$), with a p-value of 0.0475. This finding suggests that administering radiotherapy at doses below 60 Gy is associated with improved overall survival, highlighting the clinical benefit of radiotherapy in this patient population.

## Data Availability

The data used during the current study are available from the corresponding author on reasonable request.
